# Analysis of fracture-related infections from Swedish insurance claims between 2011 and 2021

**DOI:** 10.1038/s41598-023-50224-y

**Published:** 2023-12-19

**Authors:** Pendar Khalili, Staffan Tevell, Per Fischer, Nils P. Hailer, Olof Wolf

**Affiliations:** 1 Department of Surgical Sciences, Orthopedics, Uppsala University, Uppsala, Sweden; 2 Department of Orthopedic Surgery, Karlstad Hospital, Rosenborgsgatan 9, 652 30 Karlstad, Sweden; 3 Centre for Clinical Research and Education, Region Värmland, Karlstad, Sweden; 4 Department of Infectious Diseases, Karlstad Hospital, Karlstad, Sweden; 5 School of Medical Sciences, Faculty of Medicine and Health, Örebro University, Örebro, Sweden

**Keywords:** Epidemiology, Infectious-disease epidemiology

## Abstract

Fracture-related infections (FRI) pose a serious complication with an incidence of 1–2%. This study aimed to analyze compensation claims submitted to The Swedish National Patient Insurance Company (LÖF) because of FRI after closed/open reduction and internal fixation (C/ORIF) in the four most common fracture sites (proximal humerus, distal radius, hip, ankle). Patients registered in the LÖF database with a suspected FRI between 2011 and 2021 were identified by matching International Classification of Diseases and procedural codes indicative of a combination of fractures to the proximal humerus, distal radius, hip and ankle, C/ORIF and infection. Medical records were reviewed for fracture sites, pathogens and complications. Data from the Swedish Fracture Register (SFR) were extracted to estimate the proportion of reported claims to the presumed number of FRI. Of 122 FRI identified in the LÖF database, 34 were after C/ORIF in the proximal humerus, 12 in the distal radius, 28 in the hip and 48 in the ankle. LÖF compensated 111 patients (91%). Median time from C/ORIF to an FRI was 3 weeks (interquartile range 2–6), and 95% of all FRI occurred within 1 year after C/ORIF. *Staphylococcus aureus* was the most common pathogen in patients with a distal radius, hip and ankle FRI. In contrast, *Cutibacterium spp.* were the most common aetiology in FRI of the proximal humerus. The total number of fractures treated with C/ORIF in the four fracture sites registered in the SFR during 2021 was 18,711. Most of the FRI patients were diagnosed within the first year after C/ORIF, and 91% of the patients received compensation. Given an expected FRI incidence of 1–2%, our estimates with extrapolated data from the SFR indicate that < 10% of affected patients applied for compensation.

## Introduction

Reports on fracture-related infections (FRI) indicate an incidence of infection after internal fixation of closed fractures of 1–2%, rising sharply to 30% after open fractures^1^. Not surprisingly, FRI impact and burden patients and the healthcare system . The cost of care can rise substantially when post-operative infections occur, as evidenced by surgically treated distal radius fractures where costs can increase up to four-fold^2^.

The entity of FRI differs when compared to prosthetic joint infections (PJI), especially regarding the manifestation of clinical signs and symptoms, the need for fracture stability and dead space management and the concomitant presence of soft tissue or vascular injury^3^. In 2018, an international expert group submitted a consensus document on the definitions and classification of FRI^4^. Diagnostic features defining two levels of certainty (confirmatory or suggestive) were suggested, assessing wound features and tissue cultures in an algorithmic hierarchy.

Swedish legislation states that health care providers in Sweden must have insurance covering adverse events (AEs) and inform patients who sustain AEs about their right to claim compensation^5,6^. FRI can be classified as an AE; thus, they can be compensated by The Swedish National Patient Insurance Company (LÖF). For PJI, Kasina et al. published a report in 2018 suggesting a low proportion of claimants to LÖF (25%) but a high acceptance rate (96%) in a cohort comprising 441 patients with a PJI after total hip replacement^7^.

To our knowledge no studies have investigated the characteristics and proportions of affected patients filing claims for FRI. We aimed to analyze this in patients with a LÖF claim related to an FRI after closed/open reduction and internal fixation (C/ORIF) in the four most common fracture sites. We sought to describe patient demographics, acceptance rates and degrees of disability. Moreover, we studied time to FRI, distribution of pathogens and surgical treatment after an FRI contingent on the fracture site. Finally, we used data from the Swedish Fracture Register (SFR) to estimate the proportion of reported claims compared to the expected number of FRI cases.

## Methods

**Figure 1:**
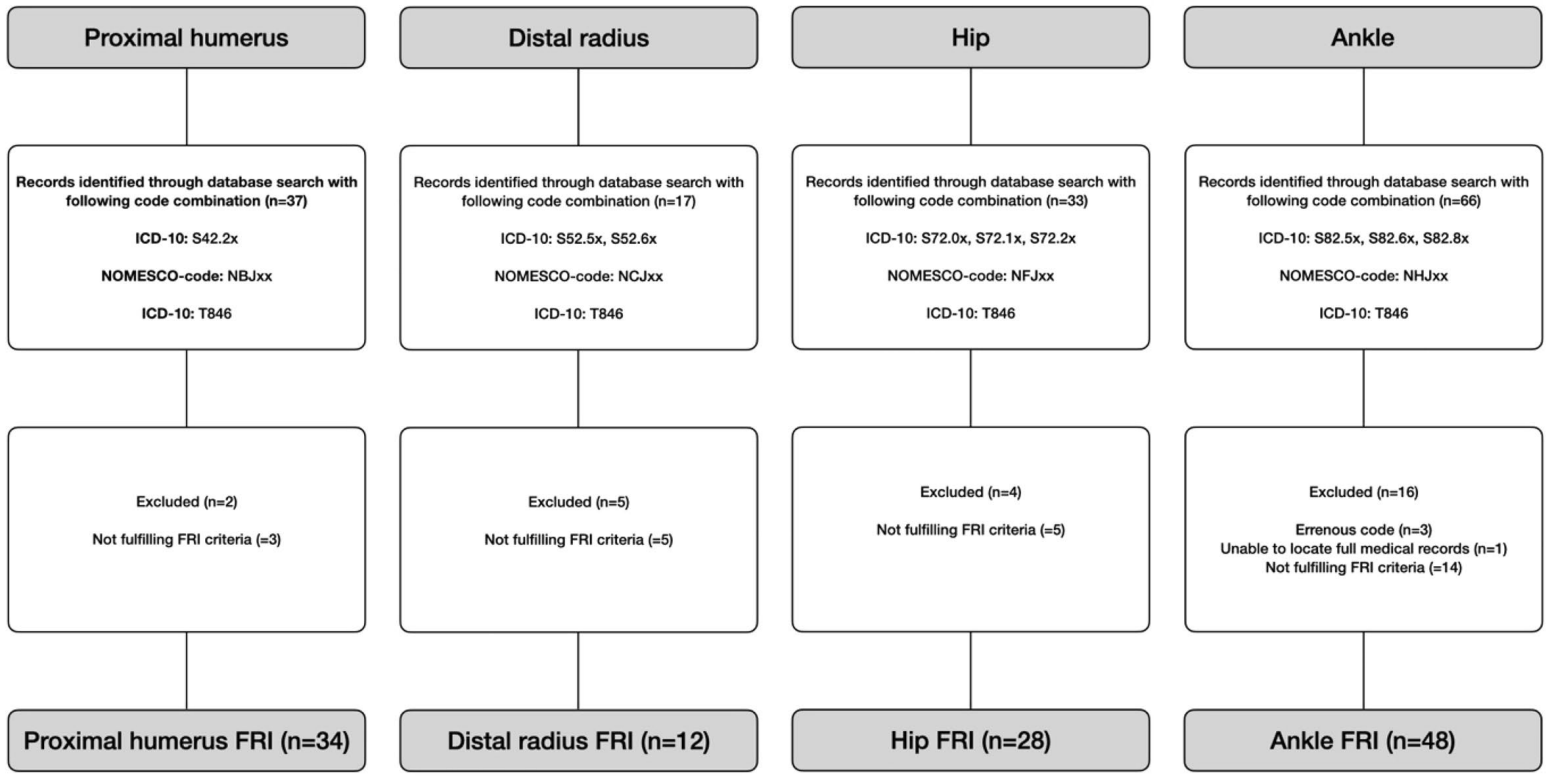
Flowchart of the study patients with an FRI after proximal humerus, distal radius, hip and ankle fractures treated with C/ORIF.

We analyzed a national retrospective cohort comprising all patients filing insurance claims submitted to LÖF between 2011 and 2021 related to an infection after C/ORIF at four fracture sites: (1) the proximal humerus, (2) the distal radius, (3) the hip and (4) the ankle. Patient claims with International Classification of Diseases (ICD)-10 codes related to these fractures in combination with a Nordic Medico-Statistical Committee (NOMESCO) fracture fixation code for osteosynthesis (excluding fracture prosthesis) and the additional ICD-10 code T84.6 (“Infection and inflammatory reaction due to an internal fixation device”) were identified in the LÖF database. All medical and insurance records were reviewed. Figure 1 describes the specific ICD-10/NOMESCO codes and the cohort extraction process.

### Patients

Patients aged ≥ 18 years with an FRI based on the updated consensus definition published by Govaert et al.^8^ were included for analysis in the final cohort. In brief, confirmatory criteria were (1) phenotypically indistinguishable pathogens identified by culture from at least two separate deep tissue/implant specimens taken during an operative intervention (including sonication fluid), (2) fistula, sinus or wound breakdown with communication to the bone or the implant and (3) purulent drainage from the wound or presence of pus during surgery. In the absence of confirmatory criteria, a suggestive criterion (e.g., erythema or elevated inflammatory markers or other typical clinical manifestations) in combination with one virulent pathogen (e.g., *Staphylococcus aureus, Pseudomonas aeruginosa*), identified by a single deep tissue/implant culture, which was also considered an FRI. This derivation is not in accordance with the original definition but has been used to identify an FRI with a high probability in a recent validation report^9^. Tissue samples taken during an operative intervention were defined as deep samples. Patients were excluded from the final cohort if they did not meet the criteria for FRI (n = 27), had incomplete medical records (n = 1) or had an erroneous ICD-10 or NOMESCO code (n = 3, one patient with ICD code T84.6 without infectious signs or symptoms and two patients with distal tibia fractures wrongly coded as ankle fractures) (Fig. 1).

### Variables

The variables sex and age were used to characterize the patient cohort. Time to FRI onset was defined as the interval from the first C/ORIF to the initial appearance of the symptoms or diagnosis according to FRI criteria. If the first clinical appearance was not defined as a confirmatory criterion, the first appearance of a suggestive criteria was considered the date of FRI onset. With subclinical FRI, in which no clinical confirmatory or suggestive criteria were present, but surgical management was still required for other reasons (e.g., pain or removal of hardware), FRI onset was determined by the date the cultures were obtained. As this study only included primary FRI, there are no relapses or recurrent infections in the present cohort. Surgical treatment was categorized into three groups: no revision surgery, one revision surgery and ≥ 2 revision surgeries. Removal of hardware was also considered a revision surgical procedure.

### Outcome variables

Compensation claims were accepted if the FRI were the reason for economic compensation. The degree of disability was extracted from the claim files from assessments performed by clinicians according to the guidelines of the Swedish Insurance Services^10^. The framework for medical invalidity and disability is based on a scale from 0 to 100%, where 100% indicates a permanent loss of any body function.

### Swedish Fracture Register

The SFR is a national quality register for all fracture types treated by orthopedic surgeons (non-surgical and surgical treatment)^11^. The register has full coverage, with all Swedish orthopedic departments (n = 54) taking part since 2021. Completeness has increased because of a stepwise introduction but differs between fracture sites. Completeness for fractures reached 60.9% in the humerus, 67.6% in the forearm, 82.9% in the femur and 67.6% in the tibia in 2021, compared to the National Patient Register (internal audit SFR). We extracted patients aged ≥ 18 years with fractures in the four chosen fracture sites, with an injury date during 2021 treated with C/ORIF within 3 weeks of the injury date. The year 2021 was selected as a representative denominator concerning completeness and incidence to approximate proportions of compensation claims. Specific ICD-10/NOMESCO codes are described in Fig. 1.

### Statistics

Counts, proportions, means with standard deviations (SDs) and medians with interquartile ranges (IQRs) were used to describe the data.

### Ethical approval

Informed consent was obtained from all subjects and/or their legal guardian(s), when patients filed a compensation claim to LÖF. The study was approved by the Swedish Ethical Review Authority (approval number 2021–04,574, date of issue 2021-10-04 and approval number 2021-06946-02, date of issue 2022-01-14) and complied with the Declaration of Helsinki.

## Results

### Demographics

We identified 122 claimants (73 women) with a mean age of 61 years, meeting the criteria of an FRI between 2011 and 2021. There were 34 patients with FRI in the proximal humerus, 12 in the distal radius, 28 in the hip and 48 in the ankle (Fig. 1). Patients with an FRI after hip fracture had a higher mean age than patients in the other fracture groups. Only three patients had an open fracture (Table 1). Definitive C/ORIF included osteosutures, external fixation, K-wires, cerclage, screws, plates, sliding hip screws and intramedullary nails (Table 2).

**Table 1:** Demographic description of the study cohort, including the number and location of the FRI. The number of patients with an FRI in each fracture site, distribution of sex and open fracture n (%), age at definitive C/ORIF (mean (± SD)) and the number (% within a group) of patients.

	Proximal humerus	Distal radius	Hip	Ankle	Total
FRI	34	12	28	48	122
Men	17 (50)	5 (42)	8 (29)	19 (40)	49 (40)
Women	17 (50)	7 (58)	20 (71)	29 (60)	73 (60)
Age	59 (± 12)	58 (± 16)	75 (± 11)	55 (± 16)	61 (± 16)
Open fracture	–	1 (8)	–	2 (4)	3 (2)
External fixation before final fracture fixation	–	–	–	4 (8)	4 (3)
External fixation as definitive treatment	–	–	–	2 (4)	2 (2)

**Table 2:** Method for definitive C/ORIF for each patient developing an FRI divided by fracture site.

Proximal humerus	34
Plate and screws	29
Osteosutures	4
Intramedullary nail	1
Distal radius	12
Plate and screws	8
K-wires	3
Plate and screws + K-wires	1
Hip	28
Intramedullary nail	13
Sliding hip screw	11
Screws/pins only	4
Ankle	48
Plate and screws	39
Plate and screws + K-wires/cerclage	4
Screws only	2
External fixation	2
K-wires/cerclage	1

### Compensation and degree of disability

Compensation from LÖF for an FRI was granted in 111 (91%) patients. Of these 111 patients, 78 (64%) also suffered a permanent disability after FRI treatment. The mean disability was 7% (± 6), with the highest degree of disability in the hip group (11% ± 9) and lowest in the ankle group (4% ± 4) (Table 5).

### Time to FRI and diagnosis

**Figure 2:**
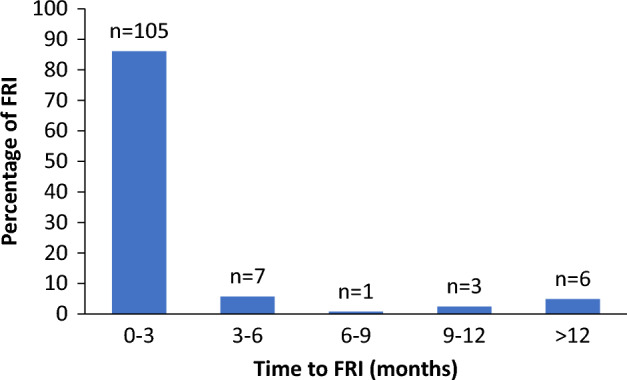
Time to FRI in a cohort of 122 patients divided into post-operative time intervals.

Median time from C/ORIF to FRI was 3 (IQR 2–6) weeks (Table 3). 86% of all FRI occurred within 3 months and 95% within 1 year (Fig. 2). 115 patients (94%) could be diagnosed using any of the confirmatory criteria, and the most common criterion in all groups was ≥ 2 deep tissue samples (86 patients, 70%) (Table 3). Seven patients (6%) were diagnosed with a suggestive criterion in combination with one virulent pathogen in a single deep tissue/implant culture (i.e., *S. aureus, n* = *6*, *P. aeruginosa, n* = *1*). They were all patients with an FRI in the ankle.

**Table 3:** Cohort and criteria description. Median (IQR) time to FRI in weeks and deep tissue cultures for each fracture site (number and % of cohort). Description of criteria for each fracture site´s FRI (n) diagnosis. Confirmatory criteria were divided into subgroups: (1) ≥ 2 deep tissue cultures, (2) wound complications, i.e., fistula, sinus or wound breakdown and (3) presence of pus, i.e., purulent drainage from the wound or presence of pus during surgery. In cases where a suggestive criterion led to an FRI diagnosis, ≥ 1 deep tissue/implant specimen culture of a significant pathogen had to be found. In cases where multiple confirmatory criteria were present, criterion (1) was considered superior to criterion (2), which in turn was deemed superior to criterion (3).

	Proximal humerus	Distal radius	Hip	Ankle	Total
FRI	34	12	28	48	122
Time to FRI, weeks	2 (2–3)	2 (1–5)	3 (2–5)	5 (2–8)	3 (2–6)
Deep tissue cultures	31 (91)	8 (67)	28 (100)	39 (81)	106(87)
Confirmatory criteria	34 (100)	12 (100)	28 (100)	41 (85)	115 (94)
≥ 2 deep tissue cultures	29 (85)	7 (58)	21 (75)	29 (60)	86 (70)
Wound complications	2 (6)	3 (25)	1 (4)	7 (15)	13 (11)
Presence of pus	3 (9)	2 (17)	6 (21)	5 (10)	16 (13)
Suggestive criteria	–	–	–	7 (15)	7 (6)

### Pathogens

Of the 106 patients with deep tissue samples, 60 had a monomicrobial FRI and 44 had a polymicrobial FRI (Table 4). Two patients had negative cultures. *S. aureus* was the most common pathogen in monomicrobial infections, whereas Coagulase-negative staphylococci (CoNS) was the most frequent among polymicrobial infections. *S. aureus* was the primary pathogen in the distal radius, hip and ankle. In polymicrobial infections *S. aureus* was most often accompanied by CoNS. In the proximal humerus FRI were most often caused by *Cutibacterium spp.,* either as a monomicrobial FRI or in combination with CoNS as a polymicrobial infection.

**Table 4:** Microbiological findings from deep tissue cultures. The number of patients in the FRI cohorts (n) with either mono- or polymicrobial infections (% of fracture site) and the number of patients with a pathogen found in cultures (% of mono- or polymicrobial subset).

	Proximal humerus	Distal radius	Hip	Ankle	Total
Total	34	12	28	48	122
Monomicrobial	13 (38)	7 (58)	13 (46)	27 (56)	60 (49)
*S. aureus*	3 (23)	5 (71)	7 (54)	15 (56)	30 (50)
CoNS	–	1 (14)	3 (23)	5 (19)	9 (15)
*Cutibacterium spp.*	6 (46)	–	–	1 (4)	7 (12)
*Streptococcus spp.*	–	–	–	1 (4)	1 (2)
*Enterococcus spp.*	1 (7)	–	1 (8)	–	2 (3)
*Enterobacterales*	3 (23)	–	–	3 (11)	6 (10)
Other	–	1 (14)	2 (15)	2 (7)	5 (8)
Polymicrobial	18 (54)	1 (8)	13 (48)	12 (24)	44 (37)
*S. aureus*	1 (6)	1 (100)	8 (62)	7 (58)	17 (39)
CoNS	13 (72)	–	7 (54)	6 (50)	26 (59)
*Cutibacterium spp.*	15 (83)	–	1 (8)	4 (33)	20 (45)
*Streptococcus spp.*	2 (11)	–	1 (8)	4 (33)	7 (16)
*Finegoldia spp.*	4 (22)	–	3 (23)	–	7 (16)
*Enterococcus spp.*	3 (17)	–	2 (15)	3 (25)	8 (18)
*Enterobacterales*	4 (22)	–	4 (31)	5 (42)	13 (30)
Other	3 (17)	1 (100)	4 (31)	4 (33)	12 (27)

### Surgical treatment of FRI

At least one revision surgery was performed in 117 patients (96%) and 76 (62%) had to undergo ≥ 2 revision surgeries. Hardware removal was conducted in 104 (85%) patients (Table 5).

**Table 5:** Surgical treatment, accepted claims and degree of disability. Treatment of FRI (n) for each fracture site, number (% of fracture site) for each treatment, compensation and assessed disability by LÖF (% of patients) and percentage degree of disability (mean (SD)). Any surgery necessary due to complications originating from the FRI was considered a revision (e.g., removal of hardware, wound revision, exchange of hardware or revision to arthroplasty).

	Proximal humerus	Distal radius	Hip	Ankle	Total
FRI	34	12	28	48	122
≥ 1 revision surgery	33 (97)	10 (97)	28 (100)	46 (96)	117 (96)
≥ 2 revision surgeries	24 (71)	6 (50)	22 (79)	24 (50)	76 (62)
Removal of hardware	27 (79)	11 (92)	23 (82)	43 (90)	104 (85)
Compensated by LÖF	33 (97)^1^	11 (92)^2^	25 (89)^3^	42 (88)^4^	111 (91)
Assessed disability	26 (76)^5^	6 (50)	17 (61)^6^	29 (60)^7^	78 (64)
Degree of disability	7 (± 4)	6 (± 7)	11 (± 9)	4 (± 4)	7 (± 6)

^1^One patient was denied compensation as compensation was already applied/received from another insurance company.

^2^One patient was denied compensation as compensation was already applied/received from another insurance company.

^3^Three patients were denied compensation; in two patients the infection was assessed to be reasonably tolerated, while one patient was denied compensation because of fistulation developed independently of the time of care.

^4^Six patients were denied compensation as the infection was assessed to be reasonably tolerated.

^5^Four patients were ultimately not regulated; hence, assessed disability and degree of disability could not always be evaluated.

^6^Five patients were ultimately not regulated; hence, assessed disability and degree of disability could not always be evaluated.

^7^Three patients were ultimately not regulated; hence, assessed disability and degree of disability could not be evaluated.

### Salvage procedures

Some patients had serious complications or needed further surgical procedures after treatment of their FRI. In the group of patients with fractures of the proximal humerus and hip 12/34 patients and 11/28 patients, respectively, underwent revision to arthroplasty. In the group with distal radius fractures 3/12 patients underwent corrective osteotomy. Another four patients in the hip fracture group eventually had a resection arthroplasty. One patient received a permanent spacer. In the group with ankle fractures 4/48 patients underwent below-knee amputations and 2/48 underwent talocrural arthrodesis.

### Swedish Fracture Register

We found that 18,711 patients were treated surgically with C/ORIF during 2021 in any of the four fracture sites: 894 patients with proximal humerus fractures, 5,645 with radius fractures, 8,171 with hip fractures and 4,001 with ankle fractures. A simple calculation with this SFR data extrapolated to the inclusion period for our LÖF cohort, 10 years, accounts for 187,110 patients. Given the lower range of incidence for FRI (i.e., 1%)^1^, the potential number of patients with an FRI during the 10-year inclusion period is 1,871.

## Discussion

Patients with FRI after C/ORIF at one of the four most common fracture sites have a high probability of receiving compensation from LÖF. Some 64% of these patients were assessed to have a permanent medical disability. Most patients were diagnosed with an FRI within the first year after C/ORIF. *S. aureus* was the most common pathogen in all locations except for the proximal humerus, and most patients underwent secondary surgical treatment. A qualified appreciation concerning the incidence of FRI, the size of our cohort and the number of surgically treated fractures show that very few patients seem to claim compensation.

### Demographics

Patients with hip FRI had higher mean age, were more likely to be female, were more likely to need ≥ 2 surgical revisions and eventually had a higher disability degree than the other three fracture sites. This observation is expected because hip fractures are often sustained at a higher age and women are in majority, mainly because of osteoporosis, impaired balance and higher comorbidity compared to patients with upper extremity and ankle fractures. The treatment of FRI in the hip often requires challenging revisions to arthroplasty, including two-stage exchange surgery, resulting in numerous reoperations, potentially leading to increased disability ^12^.

### Time to FRI and diagnosis

In our cohort 95% of FRI occurred within 1 year, with a majority within 3 months of C/ORIF (86%). The incidence peaked at about 3 weeks. Zalavras et al*.* presented similar data, with 89% of FRI developing within 1 year, although in a smaller cohort of patients (n = 46) with long-bone open fractures ^13^. Little data have been published on the time course of FRI. Previous studies have limitations, such as short follow-ups or skewed cohorts excluding patients with closed fractures ^14,15^. Our study was not dependent on follow-up time since patients apply to LÖF when their symptoms and diagnosis occur, and in the aftermath of that enabled inclusion in our cohort.

### Pathogens

The distribution of pathogens did not differ drastically in our cohort compared to previous reports^16–20^. Staphylococci, both *S. aureus* and CoNS, are the most common causative bacteria in mono- and polymicrobial infections. However, in polymicrobial FRI in our cohort other Gram-positive bacteria (e.g., *Cutibacterium spp*., streptococci and enterococci) were not uncommon. The lower number of Gram-negative aerobe and anaerobe bacteria could be linked to the low number of open fractures in our cohort^16–20^. Depending on the fracture site, there was also a difference in the distribution of pathogens, especially for the proximal humerus FRI, where *Cutibacterium spp.* was the most common causative agent. These anaerobic bacteria are colonizers, found mainly on the face, scalp, chest and back^21^. Thus, it makes sense that *Cutibacterium spp*. are not only common pathogens in post-operative infections after shoulder arthroscopy and arthroplasty^22–25^, but also in FRI after fracture surgery in the proximal humerus^26^.

### Surgical treatment of FRI

Clinical experiences and guidelines from international expert groups underline that surgical treatment with wound revision and hardware removal, if the fracture is stable, is often required in treating FRI^27^. Wound debridement is also the most feasible option to obtain deep tissue sampling for cultures and histopathology, the key to diagnosing FRI. This probably explains why almost all patients in our cohort (96%) underwent revision surgery. Emphasizing the complex course of many FRI, a large proportion (62%) of our patients underwent ≥ 2 revision surgeries, and most (85%) had hardware removed. Removal of hardware is not a routine procedure in Sweden and is only done if a post-operative complication occurs or hardware irritates the skin. A re-revision rate of 62% seems relatively high compared to the literature^28^. It could be a selection bias because our cohort hypothetically includes the most severe cases. Such patients may be more likely to file for compensation. Four patients did not undergo any surgical intervention or treatment. This finding is inconsistent with current guidelines but has been suggested to be a last resort option in patients with high comorbidity or where infection is extensive, and healing cannot be achieved^27^.

### Compensation, degree of disability and final treatment

Previous studies have investigated the relationship between orthopedic post-operative infectious complications, such as PJI and septic arthritis after anterior cruciate ligament reconstruction, and patient compensation claims to LÖF. High acceptance rates of 96% and 100% respectively were found after compensation was claimed^7,29^. Our results, with an accepted claim rate of 91%, evidence a similar trend. In the Swedish healthcare system, patients have the right to seek compensation from LÖF if they experience an avoidable injury following medical intervention. This compensation system is rooted in the principle of accountability and transparency within healthcare delivery. Rather than placing blame, the primary objective is to acknowledge and address the unintended outcomes of medical care. Experts meticulously review each case, and many FRI were deemed preventable. While compensation aids the affected patient, the primary intent of these reports is not to put blame on the care giver, but to enhance the healthcare system as a whole. Using the knowledge from these reports, LÖF also contributes to preventive projects and education in patient safety.When an FRI was determined to be unavoidable, factors such as patient non-compliance or significant co-morbidity could have played a role.

We have also shown that FRI is a severe and sometimes devastating complication, leading to demanding revision arthroplasties, arthrodesis, corrective osteotomies, chronic Girdlestone situations and, in extreme cases, amputation. These catastrophic outcomes were found in a large proportion (30%) of patients in our cohort. Through data from the accepted patient claims, we also found that many patients were assessed to have some level of permanent disability. Similar results have been published on PJI^7^. Our findings indicate a significant deterioration in the quality of life of these patients, which has also been reported elsewhere for physical function and pain interference, all of which suggest the seriousness of FRI^30–32^.

### Swedish Fracture Register and underreporting of claims

Our calculations show that < 0.1% of patients operated for a fracture with C/ORIF in one of the four investigated fracture sites file compensation claims, although 1–2% would be expected to experience an FRI^1^. Although our example with a comparison to surgical procedures in the SFR is a simple computation with multiple sources of error, it is still plausible to assume that there is a sizeable group of non-claimants with FRI. A Swedish multicenter study from 2019 strengthens this notion as data showed that 92% of the cohort with major AEs after hip replacement never filed a claim^33^.

### Strengths and limitations

The design of our study allowed the extraction of a relatively large cohort compared to other FRI studies and made it possible to review detailed data. Many studies used self-designed and inadequate definitions or the more generic Centers for Disease Control and Prevention criteria for surgical site infection^34^. However, the heterogenous nature of FRI requires consequent definitions, guidelines and treatment algorithms to offer stringency in the literature, contribute to standardized clinical reports and improve the quality of future research^9^. Thus, the FRI consensus definition of infection after C/ORIF was chosen for our study given that it has also been proven superior to previous definitions^35^. Reports of FRI are usually single-center experiences, but because of our methodology, we have included clinics from the whole country.

Our study has several limitations worth noting. Most importantly, our study is not an incidence report but merely a description of FRI patients filing compensation claims to LÖF. There could be a selection bias on the number of patient claims and why patients apply. Patients who acquire unexpected complications may be encouraged to file a claim, as opposed to patients with suboptimal conditions or high co-morbidity that may not find it reasonable to apply for insurance. The influence of the attitude and approach of the responsible clinician is also unpredictable. This could explain the relatively few patients with open fractures (n = 3) in our cohort, as FRI in this patient group cannot always be designated as an unexpected complication. The retrospective design has some shortcomings as well. For example, documenting results from tissue cultures can sometimes be incomplete. Furthermore, some FRI may have been missed because of erroneous coding. However, we believe this risk is small based on our experience reviewing material in this cohort. It is more probable to expect an overuse of this code, making it necessary for a structured analysis after the collection of medical records. Retrospectively, determining the exact time of onset for FRI is also challenging. Apart from doctors’ delay, some patients may not have contacted health care professionals at the exact time of symptom onset or may have forgotten the precise date, resulting in inaccurate documentation. We do not expect these potential delays to be large enough to substantially affect our results because patients cannot reasonably neglect infectious complications for too long. Patients with subclinical FRI (e.g., non-unions presumed to be aseptic, symptoms such as pain or stiffness) present a more serious diagnostic challenge. The diagnosis of FRI can only be determined intraoperatively in these patients, and symptoms may have been present for a long time. In our cohort, however, only one patient (in the proximal humerus group) had such an episode, reasonably not confounding our results. In contrast to some other well-designed studies using the FRI definition, we included patients with a suggestive criterion for an FRI in case of a single positive culture with a virulent pathogen (7/122 patients)^13,17–19^. Based on the results of Onsea et al*.*^9^ showing that infection is highly likely in these settings, we included these patients to ensure the possibility of capturing valuable data.

## Conclusion

A surprisingly low proportion of patients presumably affected by FRI claim compensation, but a high proportion of those who do receive reimbursements. FRI occur relatively early in the post-operative period, and the distribution of pathogens for different fracture sites is similar but with some characteristic differences. *S. aureus* was the most common pathogen in patients with an FRI of the distal radius, hip and ankle. In contrast, *Cutibacterium spp.* were the most common aetiology in FRI of the proximal humerus. Based on our results, it is reasonable to encourage patients suffering from an FRI to apply to their insurance scheme as acceptance rates are high and there is probably a respectable group of hidden numbers, at least in Sweden. Knowledge about timing and specific bacteriology could aid in diagnosis and treatment, and awareness of resulting long-term disabilities emphasizes the importance and seriousness of FRI.

## Data Availability

The data that support the findings of this study are available from LÖF but restrictions apply to the availability of these data, which were used under license for the current study, and so are not publicly available. Data are however available from the authors upon reasonable request and with permission of Swedish Ethical Review Authority, according to Swedish law on Public Access and Secrecy, chapter 21, paragraph 7 and chapter 25, paragraph 1 (https://www.riksdagen. se/sv/dokument-lagar/dokument/svensk-forfattningssamling/ofentlighets% 2D%2Doch-sekretesslag-2009400_sfs-2009-400). The datasets generated and analysed during the current study are available from the corresponding author on reasonable request and in accordance with Swedish legislation.
